# Probing Interactions in Combined Hydroxide Base Solvents for Improving Dissolution of Cellulose

**DOI:** 10.3390/polym12061310

**Published:** 2020-06-08

**Authors:** Beatrice Swensson, Anette Larsson, Merima Hasani

**Affiliations:** 1 Department of Chemistry and Chemical Engineering, Chalmers University of Technology, SE-412 96 Gothenburg, Sweden; beaswe@chalmers.se (B.S.); anette.larsson@chalmers.se (A.L.); 2 Wallenberg Wood Science Center, The Royal Institute of Technology, SE-100 44 Stockholm, Sweden

**Keywords:** cellulose, dissolution, aqueous, NaOH, hydroxide base, Kamlet-Taft, solvatochromic

## Abstract

To further understand cellulose-solvent interactions in aqueous hydroxide solutions, cellulose behavior in aqueous solutions of NaOH combined with tetramethylammonium hydroxide (TMAH) or benzyltrimethylammonium hydroxide (Triton B), as well as urea, was investigated. The rheological properties of the solutions were assessed through flow sweeps at different temperatures, and the intermolecular interactions were probed using solvatochromic dyes. The results showed that NaOH combined with TMAH had synergistic effects on cellulose dissolution and was a better solvent for cellulose than the combination of NaOH with Triton B, in spite of the superior dissolution ability of Triton B alone. This somewhat unexpected finding shows that the base pair needs to be selected with care. Interestingly, addition of urea had no significant effect on the solvatochromic parameters or dissolution capacity of solutions of Triton B but rendered improved stability of solutions containing NaOH and/or TMAH. It seems that both urea and Triton B weaken the hydrophobic assembly effect of these solutions, but urea is excluded from interacting with cellulose in the presence of Triton B. This study provides further insight into dissolution of cellulose and the possibility of utilizing combinations of hydroxide bases to achieve improved solution properties.

## 1. Introduction

There is growing interest in using cellulose as a renewable raw material for products such as hydro-/aerogels, films, and various filaments for textiles and non-woven applications, and the dissolution of cellulose is often required for these applications as well as for certain analytical methods. The dissolution of cellulose is, however, not trivial, and much research has been invested in understanding the mechanisms behind it. One solvent that has been extensively researched due to its sustainable character and viability in large-scale applications is aqueous solutions of sodium hydroxide, which can dissolve cellulose to a very limited degree at temperatures below zero and concentrations around 2 M [1]. The use of NaOH (aq) as a solvent for cellulose is interesting since it is non-toxic, cheap, and can provide fast dissolution. Its dissolution capacity, however, is low both with regard to the amount and the length of the chains that can be dissolved, and another issue is the stability of the solutions, as they gel with increasing time and temperature [2]. It has also been suggested that the cellulose might not be molecularly dissolved in NaOH, but that it aggregates in solution already at low concentrations, as investigated by scattering methods [3]. There are however conflicting reports about the state of the cellulose in NaOH (aq), which suggests that the cellulose is sensitive to the preparation method and handling of the solutions [4].

The use of additives to NaOH (aq) has been explored in order to improve NaOH (aq) as a solvent for cellulose, and urea is the additive that has gained the most attention. It has been shown that adding urea significantly improves the dissolution capacity and stability of these solutions [5]. The mechanism behind urea’s influence, however, is not fully understood. While hydrogen bonding between cellulose and urea was initially proposed, more recently weak dispersion interactions between cellulose and urea have been suggested as the reason for the observed improvement [6]. Urea and derivatives of urea have also been used as additives to improve cellulose dissolution in aqueous solutions of quaternary ammonium hydroxides (QAHs) [7,8]. The ability of aqueous QAH solutions to dissolve cellulose has been known for a long time, as seen by the patent by Leon Lilienfeld from 1924 [9]. Since then, several different QAHs have been used to dissolve cellulose in aqueous solution, such as tetrabutyl ammonium hydroxide [10,11] and tetramethyl ammonium hydroxide [12]. Quaternary ammonium bases have also been used for dissolution of cellulose in organic solvents, more on this can be found in the review by Kostag et al. [13].

Lindman, Karlström, and Stigsson have suggested that the behavior of cellulose in aqueous systems (e.g., cellulose insolubility in water) is largely governed by its hydrophobic assembly effect. The axial direction of the pyranose ring is more hydrophobic than the equatorial direction since the hydroxyl groups are all in an equatorial position. This leads to the network of hydrogen bonds in water around the hydrophobic region of the pyranose ring being broken. Consequently, the water molecules situated there suffer from fewer hydrogen bonds and might also have more restricted mobility (the enthalpic and entropic contribution to the hydrophobic assembly effect is still being debated [14,15]), leading to favorable interactions if the more hydrophobic cellulose chain regions stack and exclude water from the hydrophobic surface [16]. Based on this understanding, it has been suggested that urea weakens this hydrophobic effect by replacing water in the first solvation shell in the more hydrophobic regions. Likewise, it has been shown that increasing the hydrophobicity of the cation in the hydroxide base systems (as studied on QAHs) both increases the dissolution capacity and the stability of the solutions [17].

In our previous work on the dissolution of cellulose in water-based solvents, we discovered that combining NaOH and tetramethylammonium hydroxide (TMAH) to dissolve cellulose delayed the gelation of the solution compared to dissolving cellulose in only NaOH or TMAH [18]. This justified further investigation into the possibility of combining bases to achieve positive effects on the dissolution capacity of cellulose and on solution properties. The choice of a new base to combine with sodium hydroxide to dissolve cellulose fell to benzyltrimethylammonium hydroxide (Triton B), a base that was used to dissolve cellulose in an aqueous solution as early as in the 1930s [19]. Triton B is more hydrophobic than NaOH or TMAH (see Supplementary Information for log P coefficients) and has been reported to have good solution properties for cellulose [17]. It could, therefore, be expected to improve the solution properties of cellulose solutions when combined with sodium hydroxide. In this study, we investigated the interaction properties, dissolution capacity, and flow behavior of solutions containing NaOH combined with TMAH or Triton B, while comparing this to the effect of adding urea to NaOH (aq). In addition to the common H-acceptor interactions of OH^−^ (including a strong H-bonding and partial deprotonation of cellulose hydroxyls [20]), these bases were expected to provide different levels of stabilization through dispersion interactions of their cations.

## 2. Materials and Methods

### 2.1. Material

**Figure 1:**
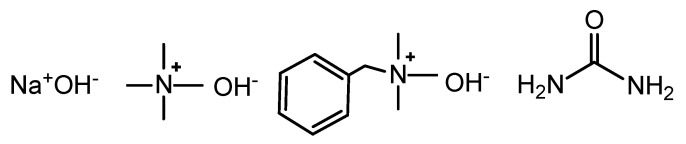
The structure of NaOH, tetramethylammonium hydroxide (TMAH), benzyltrimethylammonium hydroxide (Triton B), and urea.

Microcrystalline cellulose (MCC), Avicel PH-101 purchased from FMC BioPolymer (Philadelphia, PA, USA), a purified partially depolymerized cellulose made through the acid hydrolysis of specialty wood pulp, with a degree of polymerization of 180 (M_w_ of 29,160 g/mol) as measured with GPC-MALS (personal communication with Majid Ghasemi at Södra skogsägarnas ekonomiska förening) was used. Granulated sodium hydroxide (NaOH), known commercially as Emplura, benzyltrimethylammonium hydroxide (Triton B, see Figure 1) 40 wt.% (aq), tetramethylammonium hydroxide (TMAH, see Figure 1) 25 wt.% (aq), 4-nitroanisole(97%), 4-nitroaniline (synthesis grade) andReichardt’s dye 30 (2,6-Diphenyl-4-(2,4,6-triphenyl-1-pyridinio)phenolate, 90%) were purchased from Merck (previously Sigma-Aldrich, Darmstadt, Germany) and used as received. Urea (AnalaR NORMAPUR ACS. Reagent grade, see Figure 1) was purchased from VWR (Radnor, PA, USA) and used as received. *N,N*-diethyl-4-nitroaniline was purchased from Combi-Blocks (San Diego, CA, USA) and used as received.

### 2.2. Dissolution of Cellulose

The solvent was prepared by dissolving the desired amount of base in deionized water so that the final concentration without urea amounted to 4 mol% base or with urea 3.8 mol% base and 4.4 mol% urea. Cellulose was added to the solvent under stirring, while the solution was cooled in an ice bath and stirred for 5–15 min until the cellulose was immersed in the solvent. The temperature of the dispersion was typically around 10 °C at this point. The solution was then stored in a freezer at −20 °C for 20 min before being stirred in an ice bath for ca. five minutes to remove any ice crystals that might have formed, and to ensure a more homogeneous sample. The temperature of the solution was typically around 0 °C at this point.

### 2.3. Maximum Dissolution Limit of Cellulose

Cellulose (A [g]) was added in solution according to the method for Dissolution of cellulose (see Section 2.2), and the resulting solution was then centrifugated at 4700 rpm for 1 h at 20 °C. The supernatant was removed, and to the pellet 1 M HCl (aq) was added to ensure that the cellulose precipitated. This was then filtrated and washed with additional acid and water in a Büchner funnel. The filter cake was allowed to air dry overnight and then weighed (F [g]). The solubility in % of the added cellulose and the maximum dissolution capacity in wt.% was then calculated according to:

(1) $$\mathrm{Solubility}\;\left[\mathrm{wt}.\%\right]=\frac{100\times \left(A\;-\;F\right)}{A}$$

(2) $$\mathrm{Dissolution}\;\mathrm{capacity}\;\left[\mathrm{wt}.\%\right]=\frac{100\times \left(A\;-\;F\right)}{S\;+\;A\;-\;F}$$

where S is the weight of the solvent. Due to cellulose being a polymer and having a distribution of chain lengths, the dissolution limit for the longer chains will be reached before the dissolution limit of the shorter chains. Considering this, the maximum dissolution capacity was deemed reached when the solubility of the added cellulose was above 85 wt.%.

### 2.4. Rheology

Rotational rheology measurements were performed to investigate the flow behavior of the solutions at different cellulose concentrations and temperatures. A TA Discovery Hybrid Rheometer (HR-3), with a sandblasted 40-mm plate-plate geometry with a gap of 1 mm, was employed, and the temperature was controlled with a Peltier plate with circulating cooling liquid. Samples were measured directly after dissolution with a water-filled solvent trap and brought to the desired temperature in the rheometer without pre-shearing. Flow sweeps were conducted with shear rates from 1 to 100 [s^−1^], and increasing temperatures in series on the same sample.

The shear stress of a power-law fluid can be described by Equation (3). The flow index, n, was obtained graphically from the slope of the trendline fitted to the data in a plot of log stress vs. log shear rate.

(3) $$\tau =K\gamma^{n}$$

### 2.5. Solvatochromic Solvent Parameters

**Figure 2:**
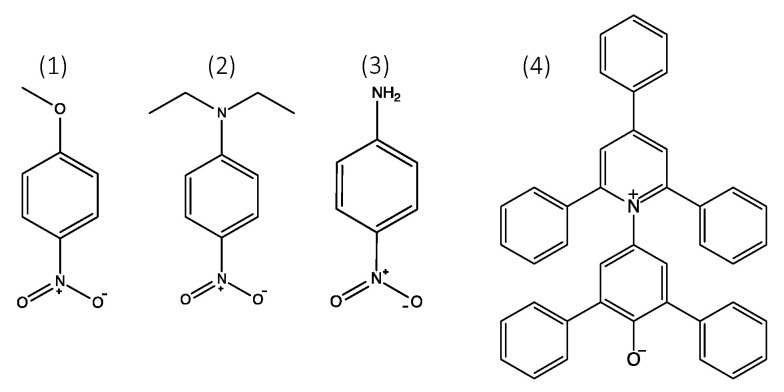
The structures of the solvatochromic dyes: (**1**) 4-nitroanisole; (**2**) *N,N*-diethyl-4-nitroaniline; (**3**) 4-nitroaniline; (**4**) Reichardt’s dye.

The dyes 4-nitroanisole, *N,N*-diethyl-4-nitroaniline, 4-nitroaniline, and Reichardt’s dye (see Figure 2) were used to obtain the parameters π, β, and α. Ca 20 mg was added to 5 mL of water and stirred for at least one hour to obtain a concentrated solution for the dyes 4-nitroanisole and 4-nitroaniline. A concentrated solution of Reichardt’s dye was prepared in the same way but in ethanol instead of water due to poor solubility. Of these concentrated solutions, ca 0.2 μL was added to cuvettes containing 1.5 mL of the sample and stirred before being measured. *N,N*-diethyl-4-nitroaniline was added directly to the samples to be measured. The instrument used for measurements at room temperature was a SPECORD 205 UV-VIS spectrophotometer from Analytik Jena (Jena, Germany), and measurements with temperature control were conducted on a Cary 4000 spectrophotometer from Agilent. Plastic (Beijing, China) disposable cuvettes with a light path length of 12.5 mm were used throughout the measurements, and water was used as the background. The maximum absorption of the peaks of interest ranged from 0.1 to 1.1. The Kamlet-Taft parameters were calculated according to Equations (4)–(7) [21,22,23].

(4) $$\pi_{1}^{}=\frac{34.12}{2.343}-\left(\frac{10,000}{2.343*\lambda_{max,1}}\right)$$

(5) $$\pi_{2}^{}=\frac{27.52}{3.182}-\left(\frac{10,000}{3.182*\lambda_{max,2}}\right)$$

(6) $$\beta =\frac{1.035\upsilon \left(\pi_{2}^{*}\right)+2.64-\upsilon \left(\beta \right)}{2.80}$$

(7) $$\alpha =\frac{\upsilon \left(RD\right)+1.873\upsilon \left(\pi_{1}^{*}\right)+74.58}{6.24}$$

This study followed the methods described in the articles by Kamlet and Taft and used the dye pairs (2) and (3) to obtain β and the pair (1) and (4) to obtain α.

### 2.6. Differential Scanning Calorimetry (DSC)

Aqueous solutions of bases were prepared as described above (see “Section 2.2 Dissolution of cellulose”), and thermoscans of the solutions were performed using a DSC 250 from TA Instruments Discovery series equipped with stainless steel pans. The solutions were cooled at a cooling rate of 10 °C/min down to −70 °C, kept at −70 °C for 5 min, and then heated up to 80 °C at a heating rate of 1 °C/min.

### 2.7. ATR FTIR

FTIR spectra of the aqueous solutions of bases were collected using a Perkin Elmer Frontier spectrophotometer (Waltham, MA, USA) equipped with an Attenuated Total Reflectance (ATR) sampling accessory, PIKE Technologies GladiATR. Samples were placed on the top of the ATR crystal and analyzed with a resolution of 4 cm^−1^ and 32 scans. Analysis of the obtained spectra was performed with Igor Pro software (8.04 version).

## 3. Results and Discussion

### 3.1. Dissolution Capacity

The dissolution limit was estimated by separation of larger, undissolved particles from the dissolved cellulose through the centrifugation of cellulose solutions (see Table 1). This limit allows for an estimation and an internal comparison within the series although it does not determine if the cellulose is molecularly dissolved. Of the estimated dissolution limits, Triton B had the highest dissolution capacity followed by TMAH and then NaOH. The dissolution capacity of TMAH was lower in this study than in our previous one due to different methods of estimating the dissolution limit [18]. Combining NaOH and TMAH to dissolve cellulose resulted in a higher dissolution capacity than for the bases alone. On the other hand, no such synergy effect was observed when NaOH was combined with Triton B; the combination of NaOH and Triton B had the ability to dissolve cellulose but to a lesser extent than pure Triton B or NaOH combined with TMAH. The addition of urea increased the dissolution capacity of solutions containing NaOH or TMAH but had no significant effect on solutions containing only Triton B. Combining NaOH and TMAH increased the dissolution capacity of solutions more than adding urea to NaOH, which in contrast, was better than combining NaOH with Triton B.

The solvents had different molecular volumes (see Table S1 in the Supplementary Information), but the results remained the same in a comparison of the maximum dissolution capacity in weight percent and in molar. Although there was most likely also a lower limit to the concentration of base required, we found that it could be as low as 3.3 mol base per AGU. Based on these findings, we continued to investigate whether the observed dissolution capacities were reflected in the flow properties of the solutions at different temperatures and whether this could be related to the interaction properties of the hydroxide solvents.

### 3.2. Flow Behaviour of the Solutions

**Figure 3:**
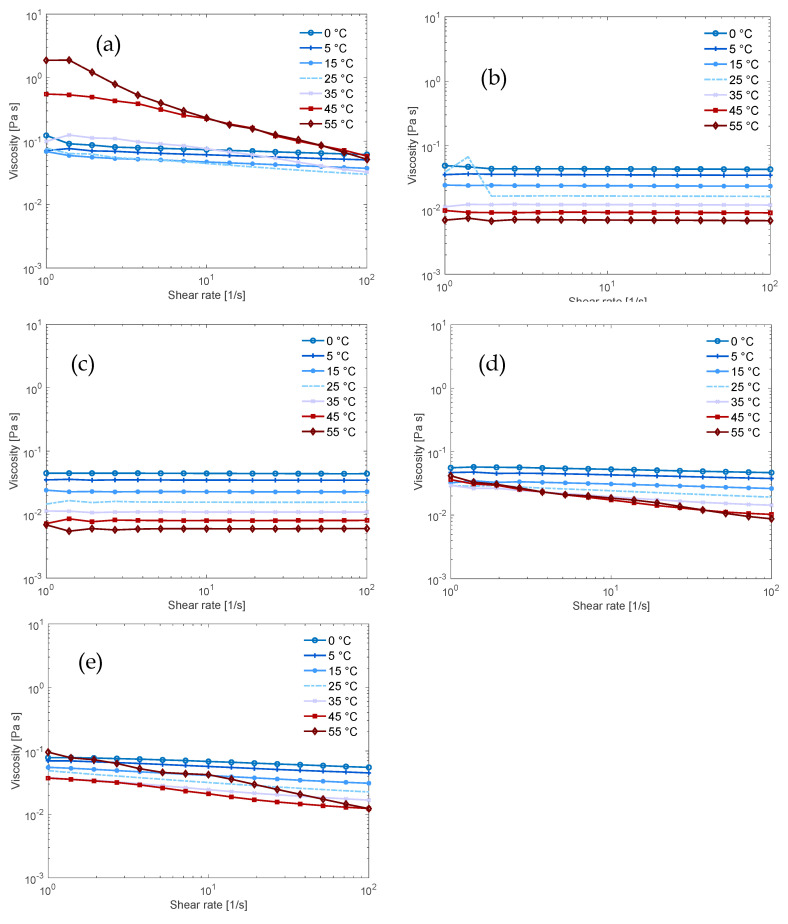
Flow sweeps of 0.36 mol% MCC in 4 mol% base (aq) solution (11:1:266 molar ratio of base:AGU:H2O) in (**a**) NaOH; (**b**) TMAH; (**c**) Triton B; (**d**) 50/50 NaOH/TMAH; (**e**) 50/50 NaOH/Triton B.

Through a series of flow sweeps at increasing temperatures, we compared the temperature stability of the solutions and received information on cellulose-solvent interactions. The NaOH (aq) solution (Figure 3a) was never fully Newtonian and this indicates that there are networks in the solution that could consist of aggregates of cellulose acting as junction points [24]. At 45 °C, in contrast, the viscosity at low shear rates increased substantially, which could be due to further aggregation and partial precipitation of the cellulose. This is in agreement with the result from the dissolution capacity since the flow sweep was conducted on a sample with ca. 3 wt.% cellulose and the dissolution limit was estimated to be 2 wt.% cellulose. For the TMAH (aq) solution, two samples of the same composition were tested, while one (Figure 3b) exhibited Newtonian behavior over the investigated temperature interval, the other was shear thinning at lower temperatures (see Figure S1 in the Supporting Information). This indicates that cellulose in TMAH (aq) has a better temperature stability than in NaOH (aq) solution but that in TMAH (aq) aggregation also occurs, even at these low cellulose concentrations.

In our previous study of the effect of combining NaOH and TMAH to dissolve cellulose, we found that combining these bases delayed gelation over time at temperatures 15–35 °C [18]. In this study, shear thinning behavior was observed as the temperature increased (Figure 3d). Shear thinning commonly indicates the formation of a transient polymer network and could in this context indicate aggregation of cellulose with increasing temperature. It could also indicate that the overlap concentration c* was reduced when the temperature increased, because the chains behaved more like stiff rods. Interestingly, this shows that the thermal stability of the combined NaOH/TMAH (aq) solution was poorer than that observed for the TMAH (aq) alone (which showed Newtonian behaviour, see Figure 3b). The observed temperature behavior is in line with the observation in our previous study that the solution in NaOH/TMAH (aq) gelled faster with increasing temperature. This could indicate an important role of the more hydrophobic cation for stabilization at higher temperatures, while the delayed gelation and improved dissolution capacity might originate from another effect. One hypothesis is that combining NaOH and TMAH disrupts the formation of the stable Na-cellulose salt (or TMAH-cellulose salt), causing the dissolved state to be more favored and to delay the gelation due to crystallization.

The Triton B (aq) solution (Figure 3c) displayed completely Newtonian behavior regardless of the applied temperature, indicating that it effectively dissolved and stabilized cellulose better than the other bases. When NaOH was combined with Triton B (Figure 3e), the flow properties deteriorated with an increase in temperature as shear thinning could be observed. Flow behavior, nevertheless, remained better than what was found for NaOH (aq) alone but poorer than for the NaOH/TMAH combination. This aligns with the results for dissolution capacity and points out a lack of synergy between Triton B and NaOH in these solutions.

**Figure 4:**
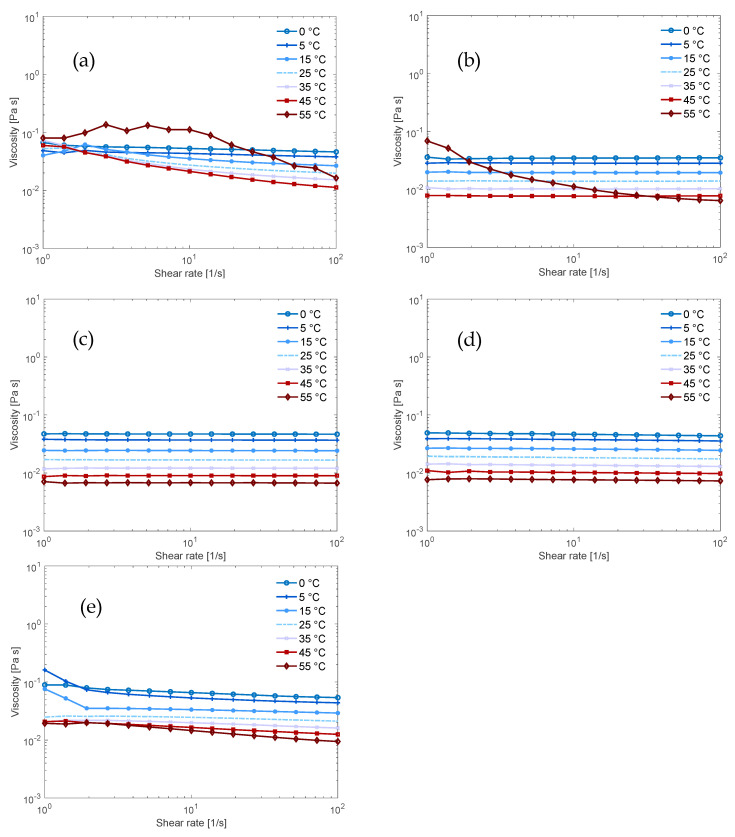
Flow sweeps of 0.34 mol% MCC in 3.8 mol% base and 4.4 mol% urea aqueous solution (11:1:266:13 molar ratio of base:AGU:H_2_O:urea) in (**a**) NaOH; (**b**) TMAH; (**c**) Triton B; (**d**) 50/50 NaOH/TMAH; (**e**) 50/50 NaOH/Triton B.

The results for the solutions that also contained urea confirm what was found for the dissolution capacity: the addition of urea enhanced the Newtonian behavior of the solutions containing NaOH and/or TMAH but did not have the same effect on the solutions containing Triton B (Figure 4a–c and also visible in the flow index in Tables S4 and S5 in the Supplementary Information). This is evident in the fact that the NaOH/TMAH/urea solution displayed Newtonian behavior over the entire temperature range (Figure 4d), thus urea improved the flow behavior while the NaOH/Triton B/urea solution had not improved as much (Figure 4e).

We also found that both urea and the more hydrophobic bases stabilized the solutions as the temperature increased. This could indicate that both urea and the more hydrophobic bases interacted with cellulose through less temperature-dependent interactions, possibly van der Waals interactions in the less polar region of the pyranose rings.

The ratios of the bases were further varied in order to observe the extent to which replacing NaOH with another base could change the properties of the solutions. Interestingly, a significant amount of the other base seemed to be required. Neither TMAH nor Triton B had any significant effect on flow properties when NaOH was replaced to a minor extent. We found that the base combination of NaOH and TMAH appeared to be optimal for the ratio of 50/50 (see Table S2 in Supplementary Information). More Triton B in the combination of NaOH and Triton B improved solution properties (see Table S3 in Supplementary Information). Note that urea is often also added to these solutions in considerable amounts.

### 3.3. Solvatochromic Parameters

To further investigate possibilities for cellulose-solvent interactions in the studied solutions, the solvatochromic parameters π, β, and α—the so-called Kamlet-Taft parameters—were determined as commonly indicative of solvent polarity/polarizability and hydrogen bonding capacity. These parameters are based on the interaction between a dye and the solvent and can indicate possible interactions between cellulose and solvents. Many dyes can be used to determine these interactions, but the absolute values obtained from different dyes should not be compared, instead trends should be compared, as demonstrated by Rani et al. [25].

#### 3.3.1. The π Parameter

**Figure 5:**
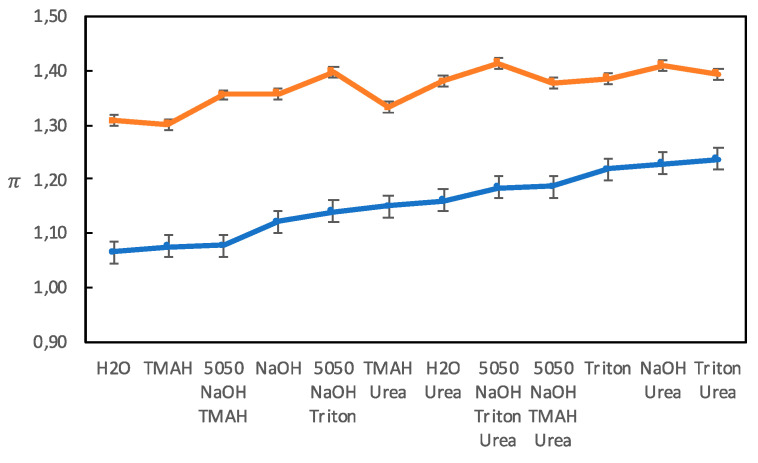
The π-values obtained with two different probes, 4-nitroanisole (blue circles) and *N,N*-diethyl-4-nitroaniline (orange squares) for the different solvents.

The π parameter measures the polarity and polarizability of a solvent, increasing with increasing polarizability and polarity with π = 0 for cyclohexane and π = 1 for dimethyl sulfoxide. According to the original method by Kamlet, Abboud, and Taft, at least seven different probes were used to measure π in order to minimize any specific interaction that could occur between a probe and a solvent. The average value of these seven π-values was then considered reliable enough to be used [21]. In most studies today, a single π-probe is used. We have used two different π probes, and as shown in Figure 5, the absolute values obtained differed, but the trends were, in general, the same.

##### Solutions without Urea

It has been shown that it is important for a good solvent to meet the amphiphilic nature of cellulose chains [16]. It is, however, difficult to use π as a measure of a solvent’s amphiphilic character since π is influenced both by polarity and polarizability. A comparison of solutions of TMAH and Triton B (see Table 2) showed that Triton B had a higher π, despite it being more hydrophobic, which must be attributed to Triton B being more polarizable due to its benzyl group with delocalized electrons. All solutions had slightly higher π-values than water, which could be due to that hydroxide ions are readily polarizable since they have an excess of electrons.

##### Solutions with Urea

The addition of urea generally increased π for both π probes (see Table 2), which can be explained by urea being more polarizable than water. The effect on the NaOH (aq) solution is in line with the findings by Wang et al., who found that adding urea to an LiOH (aq) solution increased π [6]. However, the Triton B solution appeared to be unaffected by the addition of urea, which could be indicative of a relatively strong interactions between Triton B and the π probe, excluding urea from also interacting with the probe.

#### 3.3.2. The Beta Parameter

**Figure 6:**
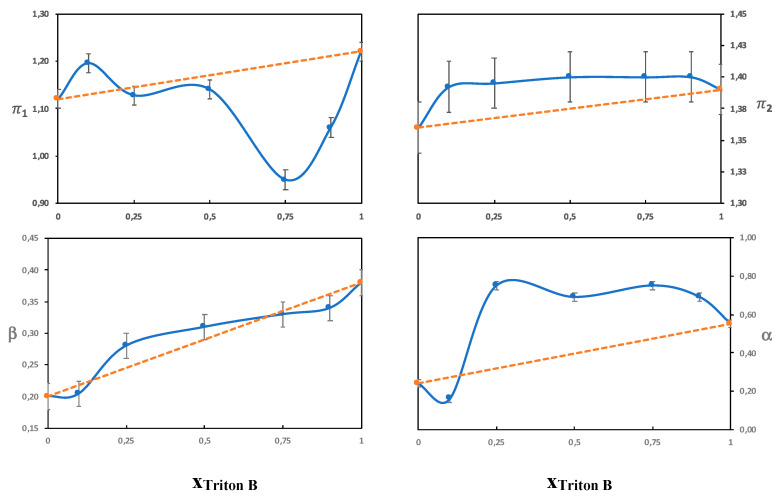
The Kamlet-Taft parameters as a function of the molar fraction of Triton B in solutions of 4 mol% NaOH/Triton B.

**Figure 7:**
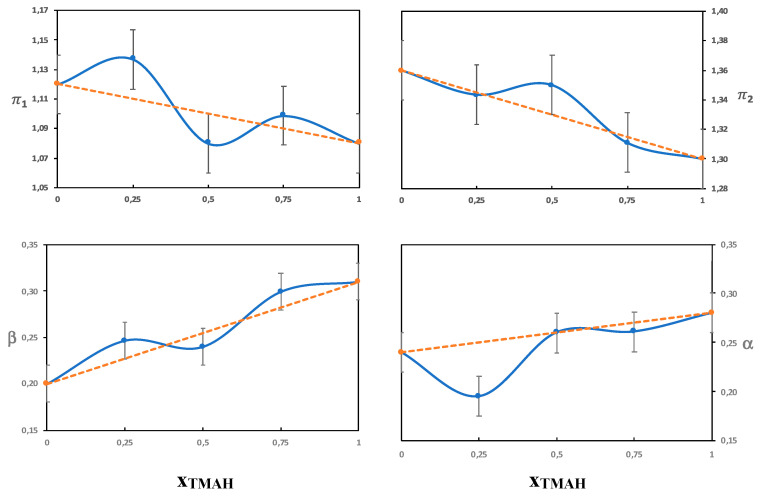
The Kamlet-Taft parameters as a function of the molar fraction of TMAH in solutions of 4 mol% NaOH/TMAH.

The β parameter measures the hydrogen bond acceptor ability of a solvent, with the reference point being the value of hexamethylphosphoramide set to 1 [22]. Both water and urea are hydrogen bond acceptors as well as hydrogen bond donors. The hydroxide ion, however, is a stronger hydrogen bond acceptor than water or urea and should thus dominate the β parameter. The solubility of cellulose in aqueous solutions of QAHs has previously been shown by Zhong et al. to increase with increasing β [26]. In line with Zhong et al., we found differences in the β-values for the different single-base solvents (Table 3) despite the fact that all the bases have the same hydroxide anion. This could originate in differences in hydration due to the different hydrophobicities of the cations. Even though the exact hydration behavior of the hydroxide ion has not been resolved, it should be affected by the presence of the counterion and local polarity. The highest β values obtained were for solutions of Triton B and might be favorable in hydrogen bonding with the hydroxyl groups on cellulose and also contribute to an enhanced deprotonation. The combined solvents had a β value corresponding to an average of the two single-base solvents, which is shown in Figure 6 and Figure 7, where the β values do not deviate much from the values of an ideal solution (that would correspond to the line in the diagram). This indicates that the observed solution properties of the combined solvents (such as delayed gelation and improved dissolution capacities) do not stem from an effect on the hydroxide’s ability to hydrogen bond.

The addition of urea to water slightly increased β. This means that urea acted as a slightly better hydrogen bond acceptor than water. When both urea and the base were present in an aqueous solution, there was no effect on β owing to the fact that it could not compete with the hydroxide ion as a hydrogen bond acceptor.

#### 3.3.3. The α Parameter

The α parameter measures the hydrogen bond donor ability of a solvent, where the value for methanol is set to 1 [23]. As previously mentioned, water and urea can act as hydrogen bond donors as evidenced by their high α values (Table 4). When NaOH is dissolved in water, α is reduced because water forms hydrogen bonds with the hydroxide ions instead of with the α probe. This shows that the anion has an indirect effect on α. None of the cations investigated in the present study have hydrogens capable of conventional hydrogen bonding, despite this, α increased in the order of Na^+^ < TMA^+^ << Triton B^+^ at 4 mol% base. Water has a high hydrogen bond donor ability, and if the cation does not contribute to the α value, then increasing the concentration of the base should lower α. The results in Table 5 show that as the concentration of the base increased, α decreased for NaOH and TMAH but not for Triton B. A possible reason for this, as discussed above, is that the more hydrophobic bases are less hydrated, which increases the possibility for Lewis acid activity since the positive charge is not as shielded. Another reason might be that the water around the hydrophobic Triton B, with the contribution of so-called dangling water with fewer hydrogen bonds (reported for hydrophobic solutes [27]), might be more prone to hydrogen bond to the alkoxide of the dye, unless its mobility is too restricted. The presence of dangling water in Triton B (aq)-containing solutions was investigated using ATR-FTIR. Indeed, an assessment of the OH-stretch band of water indicated the presence of a water fraction with a lower level of hydrogen bonding. The shoulder of the OH-band shifted towards higher wavenumbers in all Triton B-containing solutions (see Figure S11 in the Supplementary Information), and this shift is commonly attributed to an increase of the fraction of dangling water [28,29].

##### Urea

The addition of urea to the solutions decreased α, indicating that urea is a weaker hydrogen bond donor than water (the bases offered no competition as hydrogen bond donors). The solutions with Triton B were an exception, i.e., the addition of urea had no significant effect on α, which was also found for the π values. This could mean that owing to Triton B’s strong interactions attributed to lower hydration and more potential for vdWs interactions, urea was completely excluded from interacting with the probe. This correlates with the finding that the addition of urea barely increased the dissolution capacity of Triton B.

This finding can be summarized into possible interactions between cellulose and the bases; in these very basic solutions (pH ca. 14), one can expect a portion of the cellulose hydroxyl groups to be deprotonated, which would certainly have a positive effect on dissolution. Considering the polyelectrolyte effect, however, most of the hydroxyl groups can be considered to remain in their protonated state. The electrostatic interaction between a cation and a hydroxyl group would not be as strong as that observed between a cation and the zwitterionic alfa probe. This indicates that the superior dissolution power that Triton B has over NaOH or TMAH might not only be due to the hydrophobicity of the cation but also due to electrostatic interactions between the cation and cellulose.

##### On the Combinations

A comparison of the combination of NaOH and TMAH to the individual base solutions showed small differences in the measured Kamlet-Taft parameters, but overall, they were on the same levels despite clear differences in dissolution properties. Based on this finding, it seems that the improved dissolution properties do not primarily stem from an impact on polarity/polarizability or hydrogen bonding. When NaOH was combined with Triton B the behavior was quite different, i.e., the solvatochromic parameters seemed to be dominated by the properties of Triton B. This was especially evident in the high alfa value obtained and shows that Triton B probably enriches in the shell around the dye, and therefore the dye does not reflect the properties of the bulk solvent. To confirm this, we varied the ratio of NaOH/Triton B (see Figure 6) and NaOH/TMAH (see Figure 7). It was found that the NaOH/Triton B solution does not behave as an ideal solution because the values differed clearly from that of the average, as opposed to the NaOH/TMAH solution. The structure of the dyes and cellulose are very different, nevertheless, cellulose could also be expected to show a preference for a more hydrophobic base. To shed further light on the lack of synergy between NaOH and Triton B, the structure of their water solution was analyzed by DSC in order to identify hydrates of the bases and compare them to the previously studied combination NaOH/TMAH (aq).

### 3.4. DSC

In our previous study [18], we found that in a 4 mol% NaOH(aq) solution there was a NaOH hydrate with a melting temperature of −33.7 °C and ice, that melted at −9.7 °C (see Table 6). In a 4 mol% TMAH (aq) solution there was a TMAH hydrate with a melting point at −26.3 °C and ice, that melted at −17.3 °C. In a solution of 4 mol% 50/50 NaOH/TMAH (aq), NaOH and TMAH still formed hydrates with similar structure as in the single base solutions [18]. Interestingly, when NaOH was combined with Triton B, thermoscans on 50/50 NaOH/Triton B (aq) showed no formation of the otherwise observed NaOH crystalline hydrate. Only one peak could be observed, and although it is difficult to identify the structure that this originates from, this indicates that combining the two bases, NaOH and Triton, B disrupts the hydrate structure of the respective bases (probably active in cellulose-base interactions), compared to the single base solutions. This finding might explain why combining NaOH with TMAH has a synergistic effect on cellulose dissolution and solution properties, while combining NaOH with Triton B does not have the same effect, but further investigation with additional methods is required to certify this.

## 4. Conclusions

Combining bases with different properties and different advantages can have the same effect on cellulose dissolution as an additive. Adding urea to solutions of NaOH and/or TMAH, however, was found to further improve dissolution and can successfully be used together with these bases. Adding urea to Triton B did not seem to have a significant effect on cellulose dissolution, and the solvatochromic probes indicated that urea might be excluded from interacting with cellulose in the presence of Triton B. Both urea and Triton B are believed to weaken the hydrophobic effect by replacing water around the pyranose ring. The combination of NaOH with TMAH improved dissolution more than combining NaOH with Triton B. A tentative finding is that in order for a combination of hydroxide bases to have synergistic effects on cellulose dissolution, the presence of another base must still allow for the other to form the hydrates relevant for cellulose dissolution. Both urea and the more hydrophobic bases increased the temperature stability of the solutions at elevated temperatures, which was due to a larger contribution of less temperature-sensitive van der Waals interactions.

## Figures and Tables

**Table 1:** The estimated maximum dissolution capacity of microcrystalline cellulose (MCC) in the different solvents given in weight% of the total weight of the solution and in mol MCC per L of solvent. Also given is the calculated mol base per mol AGU at the dissolution limit. The results are arranged in order of increasing dissolution capacity.

Solvent (aq)	Dissolution Capacity [wt.%]	Dissolution Capacity [mol AGU/L]	Mol Base per mol AGU at the Dissolution Limit
4 mol% NaOH	2.1 ± 0.4	0.14 ± 0.03	15.9
4 mol% TMAH	2.7 ± 0.1	0.16 ± 0.01	11.2
4 mol% NaOH + Triton B (50/50)	2.9 ± 0.2	0.19 ± 0.01	9.9
4 mol% NaOH + urea	3.4 ± 0.1	0.24 ± 0.01	8.4
4 mol% NaOH + TMAH (50/50)	4.0 ± 0.3	0.26 ± 0.02	7.8
4 mol% NaOH + Triton B (50/50) + urea	4.0 ± 0.3	0.26 ± 0.02	6.5
4 mol% TMAH + urea	4.9 ± 0.1	0.32 ± 0.00	5.3
4 mol% NaOH + TMAH (50/50) + urea	6.0 ± 0.3	0.41 ± 0.02	4.4
4 mol% Triton B	6.5 ± 0.2	0.42 ± 0.01	3.9
4 mol% Triton B + urea	6.9 ± 0.2	0.47 ± 0.01	3.3

**Table 2:** The π parameters for the specified solvent and the difference in π for the same solvent with urea, obtained at room temperature.

4 mol% (aq) Solvent at RT	$\pi_{1}^{}\;\pm \;0.02$	$\pi_{2}^{}\;\pm \;0.01$	Δπ_1,urea_	Δπ_2,urea_
H_2_O	1.06	1.31	0.10	0.07
NaOH	1.12	1.36	0.11	0.05
TMAH	1.08	1.30	0.07	0.03
Triton B	1.22	1.39	0.02	0.01
50/50 NaOH/TMAH	1.08	1.35	0.11	0.02
50/50 NaOH/Triton B	1.14	1.40	0.04	0.01

**Table 3:** The β parameters for the specified solvent and the difference in β for the same solvent with urea, obtained at room temperature.

4 mol% (aq) Solvent at RT	β ± 0.02	Δβ_urea_
H_2_O	0.13	0.04
NaOH	0.20	0.01
TMAH	0.31	−0.02
Triton B	0.38	−0.01
50/50 NaOH/TMAH	0.24	0.00
50/50 NaOH/Triton B	0.31	−0.01

**Table 4:** The α parameters for the specified solvent and the difference in alfa for the same solvent with urea. The values are an average of the values obtained at 15 and 35 °C.

4 mol% (aq) Solvent	α ± 0.02	Δα_urea_
H_2_O	1.05	−0.10
NaOH	0.24	−0.11
TMAH	0.28	−0.08
Triton B	0.55	0.03
5050 NaOH TMAH	0.26	−0.05
5050 NaOH Triton B	0.69	−0.04

**Table 5:** The Kamlet-Taft parameters for increasing concentrations of base, obtained at room temperature (* values are an average of measurements at 15 and 35 °C).

Solvent at RT	$\pi_{1}^{}\;\pm \;0.02$	$\pi_{2}^{}\;\pm \;0.01$	β ± 0.02	α ± 0.02
4 mol% NaOH	1.11	1.36	0.20	0.24 *
8 mol% NaOH	1.22	1.29	0.39	0.04
4 mol% TMAH	1.06	1.30	0.31	0.28 *
6.2 mol% TMAH	1.06	1.29	0.40	0.17
4 mol% Triton B	1.20	1.39	0.38	0.55 *
6.7 mol% Triton B	1.24	1.37	0.47	0.57
4 mol% 50/50 NaOH/TMAH	1.15	1.35	0.24	0.26 *
8 mol% 50/50 NaOH/TMAH	1.09	1.30	0.40	0.09
4 mol% 50/50 NaOH/Triton B	1.16	1.40	0.31	0.69 *
8 mol% 50/50 NaOH/Triton B	1.23	1.37	0.47	0.61

**Table 6:** Melting temperature and enthalpy of peaks in 4 mol% (aq) base solutions.

Solvent	T_m,1_ °C	ΔH_1_ J/g Sample	T_m,2_ °C	ΔH_2_ J/g Sample	T_m,3_ °C	ΔH_3_ J/g Sample
NaOH *	−33.7	95.0	−9.7	170.6	–	–
TMAH *	−26.3	68.0	−17.3	27.8	–	–
Triton B	−21.2	88.5	–	–	–	–
50/50 NaOH/Triton B	−16.3	38.5	–	–	–	–
50/50 NaOH/TMAH *	−27.8	15.3	−25.1	72.2	−14.7	42.1

* previously published in [12].

## Supplementary Materials

The following are available online at https://www.mdpi.com/2073-4360/12/6/1310/s1, Partition coefficients of the quaternary ammonium hydroxide bases, Figure S1: Flow sweep of 0.36 mol% MCC in 4 mol% base (aq) solution in TMAH, Figures S2–S9: UV-visible spectra used to obtain the solvatochromic parameters, Figure S10: A picture of the alpha probe in 5 different solvents, Figure S11: The ATR-FTIR spectra of the different solvents, Table S1: The measurements used to obtain the molar of the solutions, Tables S2–S5: flow index of cellulosic solutions.
