# Supplementary material for: Probing Interactions in Combined Hydroxide Base Solvents for Improving Dissolution of Cellulose
Source: Polymers (Basel). 2020 Jun 8;12(6):1310. doi: 10.3390/polym12061310 (PMC7362248; doi:10.3390/polym12061310)
Supplement: Supplementary file 1 [file polymers-12-01310-s001.pdf]

Supplementary material to **Probing interactions in combined hydroxide base solvents for improving dissolution of cellulose**

Beatrice Swensson; beaswe@chalmers.se [1], Anette Larsson; anette.larsson@chalmers.se [1,2] and Merima Hasani; merima.hasani@chalmers.se [1,2,*]

[1] Department of Chemistry and Chemical Engineering, Chalmers University of Technology, SE-412 96 Gothenburg, Sweden 1
[2] Wallenberg Wood Science Center, The Royal Institute of Technology, SE-100 44, Stockholm, Sweden
* Correspondence: merima.hasani@chalmers.se; Tel.: +46-31-772-29-96 (SE)

Partition coefficients of the quaternary ammonium hydroxide bases in octanol/water, calculated according to the method by Wang et al.[1].

Log P of the cation of tetramethylammonium hydroxide = -0.172
Log P of the cation of benzyltrimethylammonium hydroxide (Triton B) = 2.696

[1] R. Wang, Y. Fu, and L. Lai, "A New Atom-Additive Method for Calculating Partition Coefficients," *J. Chem. Inf. Comput. Sci.*, vol. 37, no. 3, pp. 615–621, 1997.

Table S1. The measured volume of the specified solvents.

4 mol% base: 0.023 mol base in 10 ml water	Volume [ml]
NaOH	10.3
TMAH	12.5
Triton	14.0
NaOH+TMAH (50/50)	11.5
NaOH+Triton (50/50)	12.0
3.8 mol% base + 4.4 mol% urea: 0.0235 mol base + 0.026 mol urea in 10 ml water	Volume [ml]
NaOH+urea	11.5
TMAH+urea	13.5
Triton+urea	15
NaOH+TMAH (50/50) +urea	12.5
NaOH+Triton (50/50) +urea	13.5

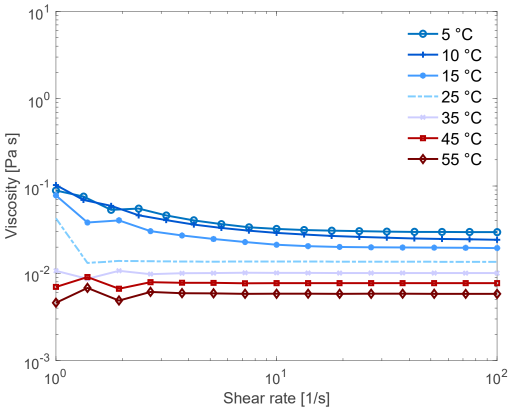

Figure S1. Flow sweep of 0.36 mol% MCC in 4 mol% base(aq) solution (11:1:266 molar ratio of base:AGU:H2O) in TMAH

Table S2-S5. The calculated flow index n obtained from the flow sweeps for different compositions of solutions as a function of temperature. In all solutions without urea the cellulose concentration was 0.36 mol% (between 2.4 and 3.0 wt.% depending on the weight of the solvent). In all solutions with urea the cellulose concentration was 0.36 mol% (between 2.15 and 2.6 wt.% depending on the weight of the solvent).

Table S2. The flow index n as a function of temperature for different compositions of NaOH/TMAH solutions.

T [°C]	n(NaOH)	n(9010)	n(7525)	n(5050)	n(2575)	n(TMAH)
0	0.89	0.76	0.81	0.95	0.96	0.98
5	0.92	0.73	0.71	0.95	0.87	0.99
15	0.89	0.67	0.68	0.94	0.85	0.99
25	0.81	0.67	0.65	0.9	0.77	0.84
35	0.71	0.59	0.63	0.84	0.73	1
45	0.48	0.52	-0.05	0.72	0.69	1
55	0.20	0.4	0.15	0.68	0.5	1

Table S3. The flow index n as a function of temperature for different compositions of NaOH/Triton solutions.

T [°C]	n(NaOH)	n(9010)	n(7525)	n(5050)	n(2575)	n(Triton)
0	0.89	0.84	0.794	0.92	0.97	1
5	0.92	0.78	0.7727	0.9	0.98	1
15	0.89	0.71	0.7033	0.87	0.8	1
25	0.81	0.66	0.6337	0.83	0.98	1
35	0.71	0.44	0.616	0.81	0.985	1
45	0.48	0.24	0.5626	0.74	0.98	1
55	0.20	-0.22	0.3577	0.57	0.98	1

Table S4. The flow index n as a function of temperature for different compositions of NaOH/TMAH solutions with urea.

T [°C]	n(NaOH urea)	n(7525)	n(5050)	n(2575)	n(TMAH urea)
0	0.94	0.95	0.95	1	1
5	0.95	0.95	0.99	1	1
15	0.85	0.85	0.97	1	1
25	0.77	0.78	0.98	1	1
35	0.68	0.74	0.96	1	1
45	0.62	0.71	1	1	1
55	0.63	0.74	0.96	1	0.52

Table S5. The flow index n as a function of temperature for different compositions of NaOH/Triton solutions with urea.

T [°C]	n(NaOH urea)	n(7525)	n(5050)	n(Triton urea)
0	0.94	0.93	0.89	1
5	0.95	0.94	0.88	1
15	0.85	0.94	0.95	1
25	0.77	0.91	0.96	1
35	0.68	0.73	0.93	1
45	0.62	0.68	0.88	1
55	0.63	0.6	0.82	1

Examples of the spectra of the solvatochromic dyes.

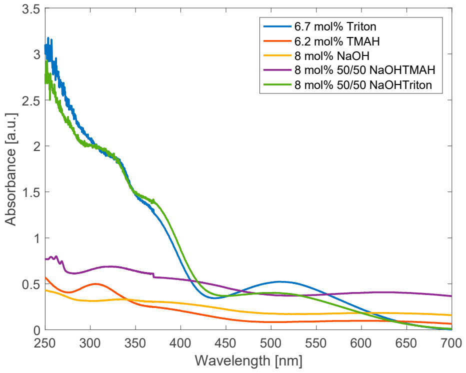

Fig. S2 UV-Vis spectra of Reichardt's dye.

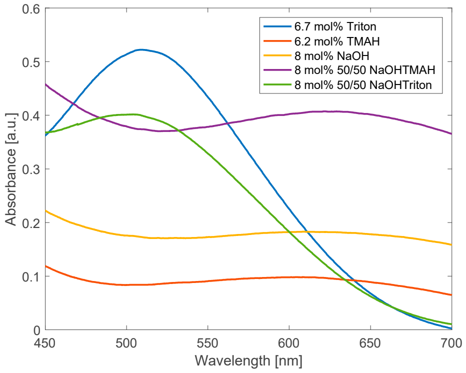

Fig. S3 UV-Vis spectra of Reichardt's dye

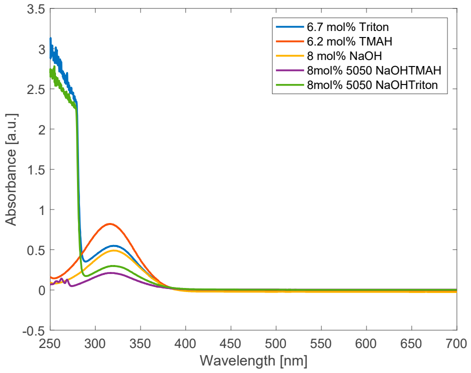

Fig. S4 UV-Vis spectra of 4-nitroanisole

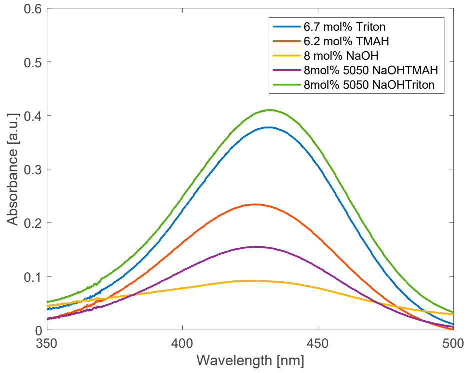

Fig. S5 UV-Vis spectra of N.N-diethyl-4-nitroaniline

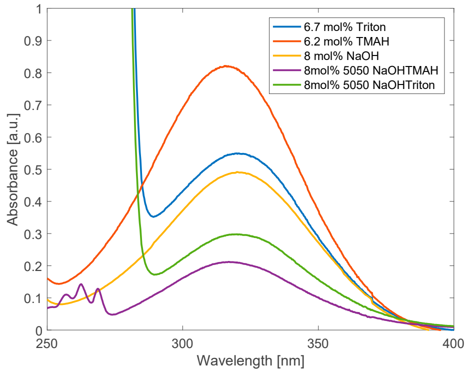

Fig. S6 UV-Vis spectra of 4-nitroanisole

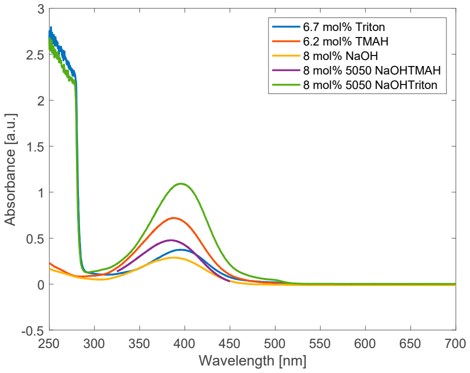

Fig. S7 UV-Vis spectra of 4-nitroaniline

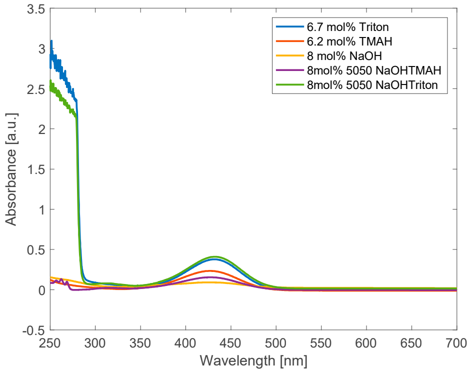

Fig. S8 UV-Vis spectra of N.N-diethyl-4-nitroaniline

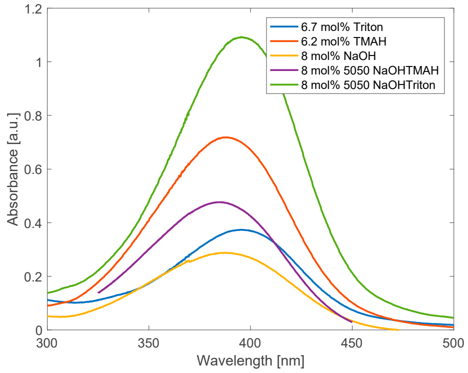

Fig. S9 UV-Vis spectra of 4-nitroaniline

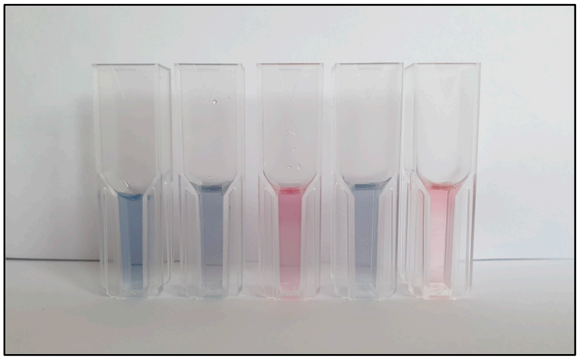

Figure S10. The α probe Reichardt's dye in 4 mol%(aq) solutions of NaOH, TMAH, Triton, 50/50 NaOH/TMAH, and 50/50 NaOH/Triton.

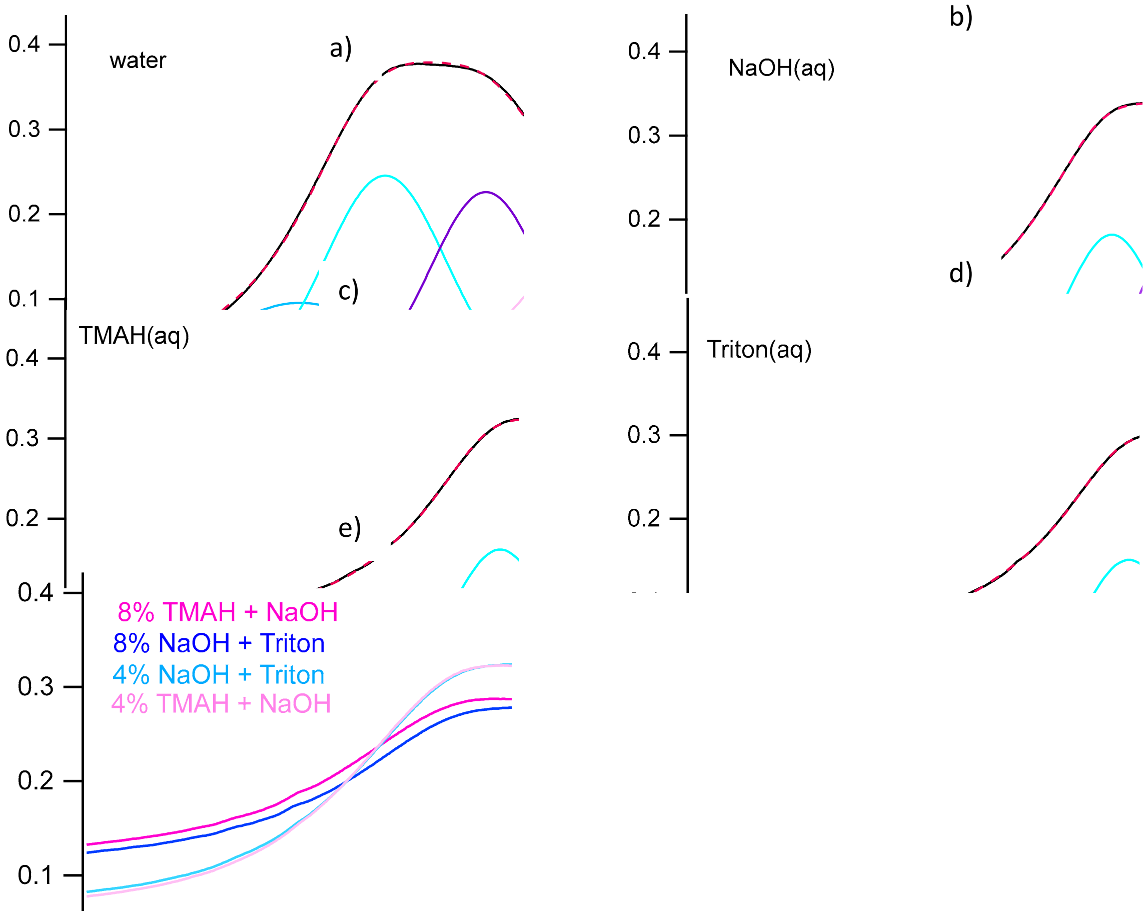

Figure S11. ATR-FTIR spectra of: a) pure water; b) 4 mol% NaOH(aq); c) 4 mol% TMAH(aq); d) 4 mol% Triton (aq) and e) 8 mol% 50/50 TMAH/NaOH, 8 mol% 50/50 Triton B/NaOH, 4 mol% 50/50 Triton/NaOH and 4 mol% 50/50 TMAH/NaOH
